# Long-Term Endurance Exercise in Humans Stimulates Cell Fusion of Myoblasts along with Fusogenic Endogenous Retroviral Genes *In Vivo*

**DOI:** 10.1371/journal.pone.0132099

**Published:** 2015-07-08

**Authors:** Sebastian Frese, Matthias Ruebner, Frank Suhr, Thierry M. Konou, Kim A. Tappe, Marco Toigo, Hans H. Jung, Christine Henke, Ruth Steigleder, Pamela L. Strissel, Hanna Huebner, Matthias W. Beckmann, Piet van der Keylen, Benedikt Schoser, Thorsten Schiffer, Laura Frese, Wilhelm Bloch, Reiner Strick

**Affiliations:** 1 Institute of Cardiovascular Research and Sport Medicine, Department of Molecular and Cellular Sport Medicine, German Sport University Cologne, Am Sportpark Muengersdorf, Cologne, Germany; 2 University Hospital Zurich, Department of Neurology, Frauenklinikstrasse, Zurich, Switzerland; 3 Institute of Human Movement Sciences and Sport, Exercise Physiology, ETH Zurich, Winterthurerstrasse, Zurich, Switzerland; 4 Friedrich-Alexander University Erlangen-Nürnberg, University-Clinic Erlangen, Department of Gynaecology and Obstetrics, Laboratory for Molecular Medicine, Erlangen, Universitaetsstrasse, Erlangen, Germany; 5 University of Zurich, Balgrist University Hospital, Department of Orthopaedics, Forchstrasse, Zurich, Switzerland; 6 Institute of Anatomy, Friedrich-Alexander University of Erlangen-Nürnberg, Krankenhausstrasse, Erlangen, Germany; 7 German Sport University Cologne, Outpatient Clinic for Sports Traumatology and Public Health Consultation, Am Sportpark Muengersdorf, Cologne, Germany; 8 University Hospital and University Zurich, Division of Surgical Research, Raemistrasse, Zurich, Switzerland; 9 The German Research Centre of Elite Sport, German Sport University Cologne, Am Sportpark Muengersdorf, Cologne, Germany; 10 Ludwig Maximilian University Munich, Department of Neurology, Friedrich Baur Institute, Ziemssenstrasse, Munich, Germany; University of Louisville School of Medicine, UNITED STATES

## Abstract

Myogenesis is defined as growth, differentiation and repair of muscles where cell fusion of myoblasts to multinucleated myofibers is one major characteristic. Other cell fusion events in humans are found with bone resorbing osteoclasts and placental syncytiotrophoblasts. No unifying gene regulation for natural cell fusions has been found. We analyzed skeletal muscle biopsies of competitive cyclists for muscle-specific attributes and expression of human endogenous retrovirus (ERV) envelope genes due to their involvement in cell fusion of osteoclasts and syncytiotrophoblasts. Comparing muscle biopsies from post- with the pre-competitive seasons a significant 2.25-fold increase of myonuclei/mm fiber, a 2.38-fold decrease of fiber area/nucleus and a 3.1-fold decrease of satellite cells (SCs) occurred. We propose that during the pre-competitive season SC proliferation occurred following with increased cell fusion during the competitive season. Expression of twenty-two envelope genes of muscle biopsies demonstrated a significant increase of putative muscle-cell fusogenic genes *Syncytin-1* and *Syncytin-3*, but also for the non-fusogenic *erv3*. Immunohistochemistry analyses showed that *Syncytin-1* mainly localized to the sarcolemma of myofibers positive for myosin heavy-chain isotypes. Cellular receptors *SLC1A4* and *SLC1A5* of Syncytin-1 showed significant decrease of expression in post-competitive muscles compared with the pre-competitive season, but only SLC1A4 protein expression localized throughout the myofiber. Erv3 protein was strongly expressed throughout the myofiber, whereas envK1-7 localized to SC nuclei and myonuclei. Syncytin-1 transcription factors, PPARγ and RXRα, showed no protein expression in the myofiber, whereas the pCREB-Ser133 activator of Syncytin-1 was enriched to SC nuclei and myonuclei. *Syncytin*-1, *Syncytin*-3, *SLC1A4* and *PAX7* gene regulations along with *MyoD1* and *myogenin* were verified during proliferating or actively-fusing human primary myoblast cell cultures, resembling muscle biopsies of cyclists. Myoblast treatment with anti-Synycytin-1 abrogated cell fusion in vitro. Our findings support functional roles for ERV envelope proteins, especially Syncytin-1, contributing to cell fusion of myotubes.

## Introduction

Cell fusions leading to multinucleated cells, like syncytiotrophoblasts during placentogenesis, bone resorbing osteoclasts and myofibers for production and repair of muscles are essential for human development. Although, all of the above are characterized by cell fusions, a unifying pathway with gene members has not been found to date. Some regulators important for myoblast fusion *in vitro* using mouse cell lines have been identified, like CD164 and Interleukin-4, as well as members of the AKT and p38MAPK pathways [1] [2] [3] [4]. Interestingly, envelope (env) genes of endogenous retroviruses (ERVs), were found essential for human trophoblast/syncytiotrophoblast fusions and were also involved in the process of multinucleated osteoclasts [5] [6]. ERVs are derived from exogenous retrovirus infected germ cells, which integrated into the genome more than 45 and less than 0.2 million years ago where some *ERV* genes produce functional proteins [7]. *Syncytin-1*, the env gene of *ERVW-1*, and *Syncytin-2* (env of *ERVFRD-1*) were found crucial for the fusion of human trophoblasts via their receptors ASCT1/2 and MFSD2a, respectively [8] [9] [10]. Human Syncytin-3 {env of *ERVP(b)*} was also shown to be fusogenic *in vitro*, but is only lowly expressed in placentae, and envV2 (ERVV-2) of Old World monkeys was also implicated fusogenic *in vitro* [11, 12].

Muscle growth is the result of complex developmental processes comprising the activity of myogenic transcription factors, cell cycle withdrawal, apoptosis resistance and myoblast fusion into myotubes. During these processes many proteins are regulated, like induction of myostatin *in vivo* and *in vitro* [13] [14], early induction of MyoD, subsequent expression of myogenin in satellite cells (SC) [15] [16] and FoxO proteins which regulate cell cycle progression and apoptosis involved in myotube fusion [17]. During myogenesis, mononuclear myoblasts differentiate into elongated myocytes and fuse to nascent myotubes to form bi- or trinucleated nascent myotubes. Additional rounds of cell fusion between myoblasts and nascent myotubes result in the formation of large, mature myotubes with hundreds or thousands of nuclei [18] [19]. Myocytes cease cell division after fetal birth, but growth as well as regeneration occurs with SC, which are derived from the embryonic dermamyotome. SCs are considered tissue-specific stem cells and are located adjacent to the myofibers of skeletal muscles, which have the ability to re-enter the cell cycle after exercise, injury or disease thereby providing new myonuclei for postnatal growth, remodeling and regeneration of muscle [20–23]. The transplantation of only 7 SCs with one myofiber generated over 100 new myofibers and thousands of myonuclei [24]. In addition, although controversial discussed, SCs have been described in the literature to possess self-renewal capabilities by symmetric expansion or asymmetric division [25]. SCs do express a variety of specific markers, including PAX7, MYF5, myogenin, c-Met and CD34 [26]. The SC markers PAX7 and PAX3 indicate an un-differentiated state, whereas myogenin positive SCs specify a differentiated state producing myonuclei [27] [28] [29].

The model organism for myoblast fusion is *Drosophila*, where multiple crucial genes for cell migration, adhesion and the initial stages of cell fusion have been revealed, for example, *Kirre*, *Rst*, *Rac1*, *Mbc* and *Sns* [30] [4]. Despite differences in muscle structure, some of these genes could also be identified as essential for myoblast fusion in mice [4]. *In vitro* analyses have been performed the mouse cell line C2C12 and also with primary muscle cells from rodents and humans. Experiments showed that remodeling actin was essential for myoblast cell fusion [31]. Similarly during formation of multinucleated osteoclasts, a unique cytoskeletal structure called the “actin ring” was essential during bone resorption [32]. It is well known that skeletal muscle fibers can reach several centimeters in length and are equipped with a high number of myonuclei [33, 34] to assure proper skeletal muscle support and maintain skeletal muscle fiber integrity. This model proposes that skeletal muscle fibers are arranged in largely independent subunits, the so-called myonuclear domains (MND) [35, 36]. The size of MNDs is strongly related to SCs, because they can fuse with adult skeletal muscle fibers due to metabolic and/or mechanical stress [37–39]. Various authors showed that resistance training was a strong stimulus to induce adaptation-processes of skeletal muscle hypertrophy [40–42].

This present study determined the cellular changes of muscle-specific attributes (e.g. number of myonuclei per muscle fiber, mean cross-sectional area of muscle fiber, mean myonuclear length (μm) and SC number) from skeletal muscle isolated from human cyclists undergoing long-term endurance exercise. With these muscle biopsies we had the opportunity to molecularly analyse and test, if specific genes, which are known to mediate cell fusion of syncytiotrophoblasts (Syncytin-1 and Syncytin-2) and osteoclasts (Syncytin-1) along with other candidate ERV genes, play a role in myoblast fusion. Therefore, we quantified the gene expression of a spectrum of 22 ERV env genes and three cellular fusion gene receptors in muscle biopsies. Furthermore, using fractionated normal human primary muscle progenitor cells from non-cyclists we performed cell culture experiments focusing on proliferation and differentiation of actively fusing myoblast cells. We demonstrate that Syncytin-1 is an essential protein involved in mediating cell fusion. Notably, the muscle attributes along with our molecular comparison showed a similarity of ERV genes essential for myoblast fusion *in vitro* and *in vivo*.

## Materials and Methods

### Subjects

Eight endurance-trained junior males with a minimum of 5 yrs competitive cycling experience volunteered for this study. The trial was performed during the competitive-season from February to October. The cyclists underwent a physical examination according to the regulations of the International Cycling Union. Their physical characteristics at the beginning of this study were (mean ± SEM): age, 17.3 ± 0.2 yrs; body mass, 69.6 ± 1.6 kg; and peak oxygen consumption (V˙O2peak), 65.2 ± 2.0 ml•kg–1•min–1. For the quantitative determination of serum levels of free testosterone (pg•ml–1) radioimmunoassay test kits were used (Beckman Coulter, Krefeld, Germany) and for the levels of estradiol (pg•ml–1) chemiluminescence immunoassay kits with an Architect i1000SR immunoassay module (Abbott, Ludwigshafen, Germany) were used. All serum samples were analyzed in duplicate and the mean was used for statistical analysis. All participants and their guardians were informed about the experimental protocol and associated risks before their written informed consent was obtained. The study was approved by the Institutional Ethics Committee of the German Sport University Cologne, Germany, and conducted in accordance with the Declaration of Helsinki.

### Testing procedures, training and racing

After a 3-month winter pre-competitive season (November to January) consisting of sport-specific base training (long slow distance) and several days before the start of the competitive-season (February to October), all cyclists underwent blood profiling, an incremental exercise test and a muscle biopsy 3 days later (referred as pre-competitive season samples). The participants were requested to keep a record of their daily training and competition data quantified in terms of volume and intensity by heart rate (Polar S710i, Polar, Kempele, Finland). The time spent in cyclists’ individual training zones was documented in a modified classification scheme for physical activity based on relative exercise intensities providing a moderate (< 70% of V˙O2peak), hard (70–90% of V˙O2peak) and very hard (> 90% of V˙O2peak) training zone [43]. During the pre-competitive season and competitive-season the subjects maintained their regular exercise training and competition program. One week after the end of competitive-season all subjects were tested again for blood profiling, an exercise test and a muscle biopsy (referred as the post-competitive season samples). Body and leg muscle mass were recorded using bioelectrical impedance analyser (BC-418, Tanita Corporation, Tokyo, Japan). Afterwards all subjects performed an incremental exercise test to volitional exhaustion in order to assess V˙O2peak and peak power output (Wpeak) on an electromagnetically braked cycle ergometer (SRM-Ergometer, Schoberer Rad Messtechnik, Jülich, Germany). Gas exchange data were determined with an open-circuit breath-by-breath spirograph (nSpire Health, ZAN600USB, Oberthulba, Germany) throughout the testing as previously described [44]. V˙O2peak was recorded as the highest V˙O2 value observed during the test [45]. The incremental cycling test started at 100 W, increased by 40 W every 5 min at constant cadences between 80–90 r•min–1 throughout the test.

### Muscle biopsies

Muscle samples (60–95 mg) were obtained from the *vastus lateralis* muscle at one-third (± 2 cm) of the distance between the patella and anterior superior iliac spine using a 5 mm Bergstrom biopsy needle [46]. In the pre-competitive season and post-competitive season, multiple biopsy samples were obtained 3 days after the incremental exercise test of each participant. After extraction from the leg, muscle biopsy samples were embedded in Tissue-Tek (Sakura Finetek, Zoeterwoude, Netherlands), immediately frozen in liquid nitrogen-cooled isopentane and stored at –80°C.

### Histology

For the analysis of muscle fiber classification, Adenosinetriphosphatase (ATPase)-staining was performed on serial cryocut cross-sections (7μm) as previously described [47]. Muscle fiber phenotypes were matched in MyHC type I and type II fibers including IIA and IIX fibers. For muscle fiber type distribution, a mean total of 325 ± 125 muscle fibers were analysed for each participant, taken from sections at different depths of the muscle. The mean fiber type-specific diameter was determined on ATPase-stained sections using Scion Image (NIH, Bethesda, MD) calculating the ellipse minor axis [48]. To determine the number of myonuclei inside the muscle fiber, serial cryocut cross-sections (7μm) were stained with Mayer’s haematoxylin and counterstained with eosin. The cross-sections were examined under a Zeiss Observer microscope (Jena, Germany). The muscle fiber composition and the number of nuclei per muscle fiber (average of 141 fibers, range 80–162 fibers) were investigated using the PALM Robo V4 image analysis software (Zeiss, Jena, Germany). The mean cross-sectional area was determined in the same fibers using Scion Image (NIH, Bethesda, MD). For the calculation of the length of myonuclei, a range of 260–325 nuclei were quantified from serial sections from different depths of the vastus lateralis. Analyses were performed for each participant.

### Myonuclear domains

The myonuclear domain (MND) was calculated as the number of myonuclei in a muscle fiber segment X = (NxL) / (d + l) [49], with (N) as the number of myonuclei counted in a cross-section of a particular fiber profile, (L) as the desired length of the fiber segment set at 1 mm [34] [36], (d) as the thickness of the cryostat cut cross-section, and (l) as the average length of a muscle nucleus. Other parameters were used from the morphometric analyses obtained from HE-stained sections. The volume of cytoplasm (μm^3^) per myonucleus (Y) was assessed with: Y = (CxL) / X [36], with (C) as the quantified cross-sectional area of a muscle fiber profile (determined from HE-stained sections), (L) as the length of the segment (set as 1mm), and (X) as the number of myonuclei per fiber segment of the same profile as calculated by the formula above.

### Immunofluorescence and Immunohistochemistry

PAX7 and myogenin immunolocalization was performed on serial cryocut muscle cross-sections (7μm), postfixed with 4% paraformaldehyde. The slices were oxidized for 30 min at room temperature with 3% H_2_O_2_, treated with Triton-X100 and blocked with 5% BSA. Slides were incubated overnight at 4°C with primary antibodies against PAX7 (1:400) or myogenin (1:500) (both from Developmental Studies Hybridoma Bank, Iowa City, IA) and afterwards incubated with a secondary antibody (biotinylated goat anti-mouse IgG, 1:400; Dako, Glostrup, DK), streptavidin-biotinylated horseradish peroxidase complex (1:150; Amersham, Freiburg, Germany) and DAB (3,3`-diaminobenzidine-HCl, 0.1 M, pH 7.4). The SCs were counterstained with methyl green and the sections were coverslipped with Entellan (Merck, Darmstadt, Germany). For the quantification of PAX7 and myogenin a digital camera (AVT Horn, Aalen, Germany) coupled to an Axio Observer microscope (Zeiss, Jena, Germany) was used at 400x magnification. To assess muscle fiber SC content, a mean total of 192 ± 33 muscle fibers were analysed for each participant using the PALM Robo V4 image analysis software (Zeiss, Jena, Germany). To determine the localization of PAX7 positive (+) SCs, serial cryocut muscle cross-sections (7μm) were post-fixed with acetone, treated with Triton-X100 and blocked with 5% BSA. Slides were double-stained with antibodies directed against PAX7 (1:500) visualized with biotinylated goat anti-mouse IgG (1:400) labeled with streptavidin conjugated Alexa Fluor 555 (1:400; Molecular Probes, Darmstadt, Germany), and laminin (1:800; Sigma, Missouri, USA) with Cy2 conjugated goat anti-rabbit IgG (1:400; Jackson ImmunoResearch, Suffolk, UK). Myonuclei were counterstained with DRAQ5 (1:1,000; Axxora, Lörrach, Germany) and the sections were coverslipped with Aqua-Poly/Mount (Polysciences, Eppelheim, Germany). For all staining procedures negative controls were generated by omitting the primary antibodies. Images were digitally captured at 200x magnifications using a confocal laser scanning microscope (LSM 510 Meta, Zeiss, Jena, Germany) and processed using LSM Image software. The localization of MyHC-I and MyHC-II proteins in the same human muscle biopsies were done with mouse monoclonal antibodies directed for MyHC-I (1:100; A4.840) and MyHC-IIA and –IIB (1:80; BF-35) from Developmental Studies Hybridoma Bank, University of Iowa, USA. The detection of ERV env proteins, their receptors and transcription factors, muscle cryosections were treated as above and incubated with the following antibodies: Syncytin-1 (1:200; Imgenex, San Diego, USA), Syncytin-2 (1:200; abcam, Cambridge, UK) erv3 (1:1,000; everest biotech, Oxfordshire, UK), envK (1:50; USBiological, Swampscott, USA), SLC1A4 (1:200; Aviva Systems Biology, San Diego, USA), SLCA5 (1:200; Cell Signaling, Frankfurt, Germany), MFSD2 (1:200; antibodies online, Atlanta, USA), pCREB-Ser133 (1:1,000; Millipore,Temecula, USA), PPARγ (1:200; USBiological), RXRα (1:50; antibodies online) and pan cytokeratin (clone 80, 1:500; Kamiya biomedical, Seattle, USA) using the LSAB+HRP kit (Dako, Hamburg, Germany) and Hematoxylin according to the manufacturer’s instructions, [11] [50]. Normal human placental control tissues (third trimester) were incubated with anti-Syncytin-1 (1:200; Imgenex, San Diego, USA) and used as a positive control for comparison with human muscle biopsies hybridized with anti-Syncytin-1. For semi-quantification of Syncytin-1 protein, 10 independent fields of equal size from IHC muscle tissue sections were measured for signal intensities using ImageJ (http://imagej.nih.gov). Syncytin-1 protein signal intensity was then correlated with the specific MyHC-I, MyHC-IIA and MyHC-IIX myofiber type.

### Primary human myoblast cultivation

Human muscle biopsies isolated from the vastus lateralis muscle of control participants were used for myoblast isolation and cell cultures. Biopsies were obtained with Institutional Ethics Board approval from 2 healthy donors (Ludwig Maximillian’s University of Munich, Germany). Isolated myoblast preparations isolated from controls consisted of a homogenous cell population and was confirmed to be of satellite origin, using an antibody against human neuronal cell adhesion molecule (clone 123C3; Monosan, Uden, The Netherlands) according to Faenza et al. [51]. For maintaining myoblasts in culture F10 medium (Gibco-BRL, Germany) containing 15% fetal bovine serum (FBS), 5% defined supplemented calf serum (Hyclone Laboratories, South Logan, UT, USA) and 1% penicillin/streptomycin (Sigma, St. Louis, MO, USA) was used. For specific cell culture experiments either growth medium (GM) or a specific differentiation media (DM) was used. Myoblasts grown in GM (PromoCell, Heidelberg, Germany) was supplemented with 10% (v/v) fetal calf serum (Lofer, Austria), 1.5% (v/v) 100x Glutamax, and 50 μg/ml gentamicin. The myoblast GM was changed every 2 days. To initiate differentiation, the myoblasts were grown to 80% confluency in growth medium (GM) and then was replaced with DM (DMEM; Gibco-BRL) with 2% horse serum (HS) and 0.01 M insulin according to [52]. The DM was changed daily. In addition, myoblasts were grown for up to 4 days in 40μM Forskolin (Sigma-Aldrich) in GM or DM for comparison. Cell culture experiments were also designed to block Syncytin-1 protein at the cellular membrane. Primary myoblast cultures were grown in GM on coverslips coated with Laminin protein extracted from Engelbreth-Holm-Swarm murine sarcoma basement membrane (10μg/ml) (Sigma) until cells were 80% confluent (n = 3). Myoblasts were then incubated in DM media to initiate myotube differentiation (day 1) with no or with the addition of 1μg/ml anti-Syncytin-1 polyclonal antibody (Imgenex, San Diego, USA). The addition of anti-Syncytin-1 was then added on day 2 (1μg/ml) and day 3 (0.5μg/ml) in DM. On day 4 the muscle cells were fixed in 4% paraformaldehyde (PFA) and processed according to the fluorescence staining protocol below.

### RNA isolation, cDNA and Semiquantitative and quantitative real time PCR (qPCR)

RNA, cDNA and qPCR was made according to our previous publications [50, 53]. QPCR of 22 different human ERV envelope genes (Syncytin-1, -2, -3 (envP(b)), erv3, envK1-7, envV1, envV2, envE, envH1-3, envRb, envT, envFc1, envFc2, envW2 were performed using primers and PCR [53] [50]. Semiquantitative PCR was performed for the receptors SLC1A4 (TF: 5’ TGAATCAGAAGGCAACAAAGAA; BR: 5’ GATGTCTCCTCCTCAGACTTGC), SLC1A5 (TF: 5’ CTTCGTAAAGATCATCACCATCC; BR: 5’ ATGATGGCCAGAGTGAGGAC), the cell type specific genes PAX7 (TF: 5’ ACCCACTACCCAGACATATACACC; BR: 5’ TTACTGAACCAGACCTGCACAC), MyoD1 (TF: 5’ ACTTCTATGACGACCCGTGTTT; BR: 5’ GAGTGCTCTTCGGGTTTCAG), Myogenin (TF: 5’ GTGTGTAAGAGGAAGTCGGTGTC; BR: 5’ GAAGGCCTCATTCACCTTCTT) and S100A4 (TF: 5’ GCTCAACAAGTCAGAACTAAAGGAG; BR: 5’ CTTCTGGAAAGCAGCTTCATC).

### Fluorescence imaging of primary cells

Human primary myoblasts were cultured in DM or GM for up to 96 h as described above and cells were fixed using 4% PFA. The localization of F-actin was determined using Phalloidin Alexa488 (Molecular Probes, Life Tech., Darmstadt, Germany), the cell membrane was stained with wheat germ agglutinin conjugated with Alexa594 and the nuclei with Hoechst 33342 according to [11]. Representative photos were made using the confocal microscope Nikon Eclipse E1000-M or using an Olympus BX-51 fluorescent microscope (Olympus, Hamburg, Germany) equipped with an F-View II CCD camera (Soft Imaging System, Stuttgart).

### Statistical analyses

Data are presented as means ± SEM. Data was analyzed using the Kolmogorov-Smirnov tests to determine the equality of samples for comparison. Differences in muscle and hormone parameters between pre- and post-competitive season were analyzed using paired (2-tailed) t-tests. Gene expressions were analyzed using Mann-Whitney-U test. Relationships between changes in exercise volume and/ among changes in muscle parameters were described using Pearson product-moment correlation coefficient. Statistical significance was set at P < 0.05. All statistical tests were processed using the Statistica software (StatSoft Inc., Tulsa, OK, USA) and SPSS 21 (IBM, New York, USA).

## Results

**Fig 1:**
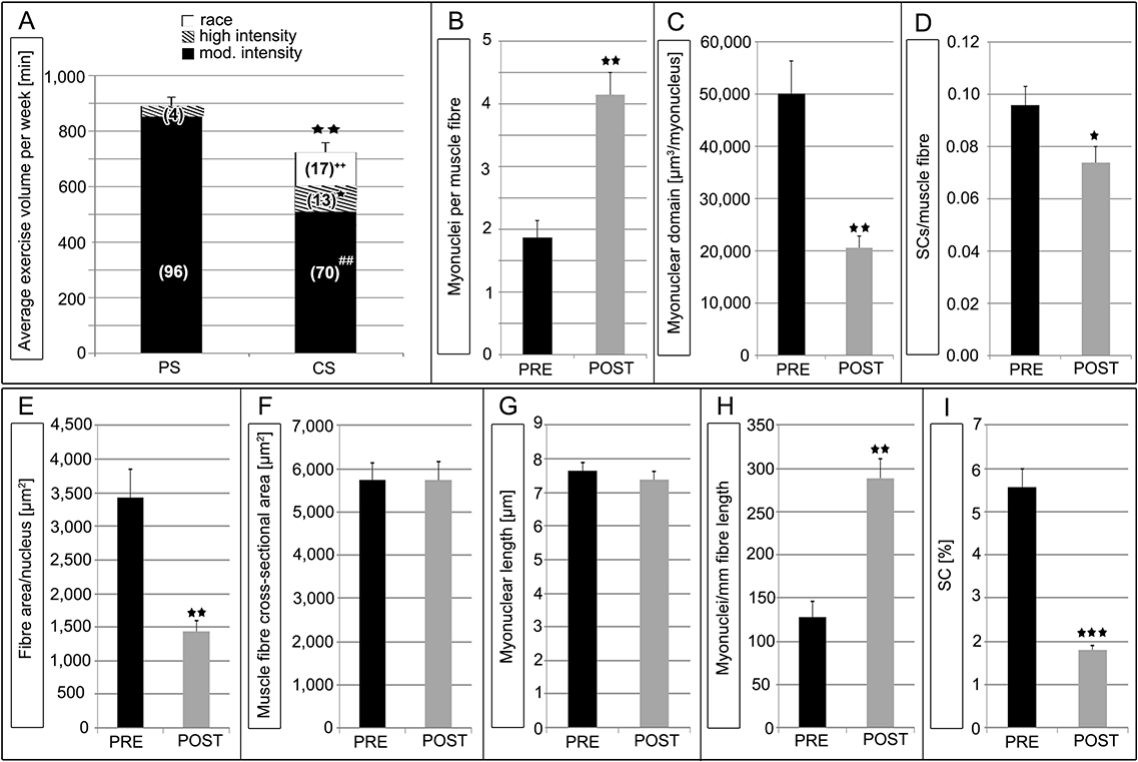
Cyclists (n = 8) were evaluated for: A) average exercise volume per week (min) throughout pre-competitive season (PS) and competitive-season (CS), B) myonuclei per muscle fibre, C) myonuclear domain (μm^3^/myonucleus) and D) SCs/muscle fibre after pre-competitive season (PRE) and post-competitive season (POST). * = P < 0.05, ** = P < 0.01, and ##, **++** = P < 0.001 (A: ++, ## indicate differences from corresponding pre-competitive season training zone and race time). Percentage of total time spent training and race in corresponding season is shown in parentheses.

The cyclists’ average weekly exercise volume (in minutes) spent at training and racing throughout the three months of pre-competitive season and the following 8 month competitive-season were analyzed. A significant decrease of the average weekly total exercise volume in the competitive-season compared to pre-competitive season was found. This was most likely due to a relative reduction of the cyclists time spent training at moderate intensity, whereas a significant increase in average weekly time spent training at high intensity and racing was during the competitive-season (Fig 1A). A significant 2.22-fold increase of myonuclei per muscle fiber was found at the post-competitive compared to the pre-competitive season (Fig 1B) whereas the mean cross-sectional area of muscle fiber remained unchanged (Table 1). Conversely the fiber area (μm^2^) per nucleus was significantly decreased in the post-competitive season by 2.4-fold compared to the pre-competitive season (P < 0.01) and without any variation in the mean myonuclear length (μm) (Table 1). A significant decrease of the myonuclear domain (μm^3^/myonucleus) and a significant increase of myonuclei per millimeter fibre length (myonuclei/mm fibre length) from the pre-competitive season to the post-competitive season were also observed (Fig 1C and Table 1). Correlation analyses revealed a significant relationship between the change in myonuclear number and increase in exercise intensity (high intensity training and racing) related from pre- to post-competitive season (r = 0.71; P < 0.05). Comparing single measurements from V’O2peak with fiber types and leg muscle masses of the cyclists between pre- and post-competitive season no significant changes were noticed (Table 2).

**Table 1:** Myonuclear and satellite cell (SC) characteristics in human vastus lateralis muscle before (PRE) and after (POST) competitive-season.

	PRE	POST
Fibre area/nucleus (μm^2^)	3,421 ± 426	1,438 ± 150**
Muscle fibre cross-sectional area (μm^2^)	5,739 ± 393	5,731 ± 443
Myonuclear length (μm)	7.65 ± 0.25	7.39 ± 0.23
Myonuclei/mm fibre length	127.70 ± 18.92	287.80 ± 23.00**
SC %	5.57 ± 0.43	1.79 ± 0.10***

Data presented as mean ± SEM. POST significantly different from PRE as

** *P* < 0.01 and

*** *P* < 0.001.

**Table 2:** Physiological and muscle fibre characteristics in cyclists before (PRE) and after (POST) competitive-season.

analysis	PRE	POST
Wpeak {W}	344.5 ± 12.4	331.5 ± 11.8
V’O2peak {l•min–1}	4.55 ± 0.20	4.98 ± 0.23
Mass {kg}	69.6 ± 1.6	70.9 ± 1.7
Hematocrit {%]	41.7 ± 0.7	42.0 ± 0.9
Hemoglobin {g•dL–1}	14.2 ± 0.3	14.1 ± 0.5
Free testosterone {pg•ml–1}	11.0 ± 1.6	10.3 ± 1.4
Estradiol {pg•ml–1}	10.1 ± 2.4	12.3 ± 3.0
Fibre type I {%}	63.5 ± 1.7	56.1 ± 4.0
Fibre type II {%}	36.5 ± 1.6	43.9 ± 4.0
Fibre type I {μm} ^	67.97 ± 2.80	63.65 ± 2.53
Fibre type IIA {μm} ^	67.89 ± 3.31	62.38 ± 2.21
Fibre type IIx {μm} ^	63.58 ± 1.67	63.33 ± 2.13
Leg muscle mass {kg}	21.0 ± 0.34	21.1 ± 0.32

Data presented as mean ± SEM. All differences between PRE and POST were not significant (P > 0.05). Mean values of Hematocrit and hemoglobin levels were measured using Sysmex system (KX-21N, Sysmex, Kobe, Japan).

^ = in ellipse minor axis.

**Fig 2:**
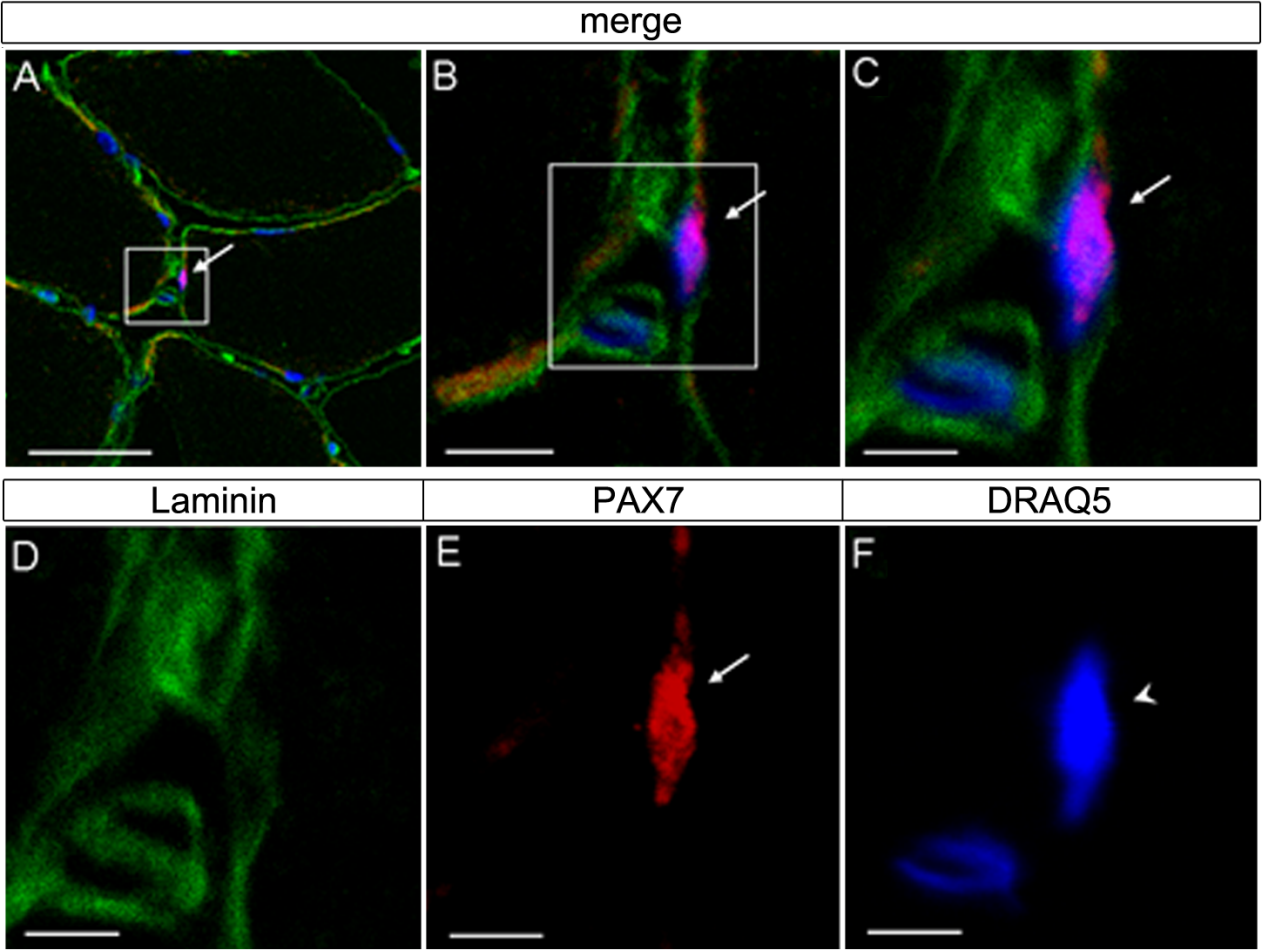
Immunostaining of serial cryocut cross-sections in *vastus lateralis* muscle of cyclist after the pre-competitive season. (A) Muscle fibers are shown, where one area is viewed at a higher magnification (white box) in (B) and (C); (D, E, F) co-immunolocalization of Laminin (green), PAX7 (red) and myonuclei (arrowhead) counterstained with DRAQ5 (blue); the marked area in (A-C) represents the same area as shown in (D–F); SCs (arrows) are indicated. (C) Note that PAX7 positive SC is located between the sarcolemma and the basal lamina of the muscle fibre. Bars: 50 μm (A), 10 μm (B) and 5 μm (C–F).

Additionally, in order to determine PAX7(+) SCs and their localisation in muscle biopsies of the cyclists before the competitive-season, an antibody specific for laminin was used to label the fiber basement membrane. SCs were located between the basal lamina and plasma membrane of the skeletal muscle fiber as previously described [20] [16]. A representative localization of PAX7(+) SCs is shown in Fig 2. Immunostaining for the SC marker myogenin was negative, however myonuclei counterstained with DRAQ5 showed co-localization with PAX7(+) nuclei of SC, indicating an undifferentiated state (Fig 2 and data not shown). Regarding the SC content, a significant decrease of PAX7(+) SCs per muscle fiber (SCS/muscle fibre) as well of the number of SCs as a percentage of the total number of nuclei (SC %) in the post-competitive compared to the pre-competitive season were identified (Fig 1D and Table 1). A relationship was also determined between the change in myonuclear number and change in SCs/fiber (r = –0.91; P < 0.01) throughout the post-competitive-season.

**Fig 3:**
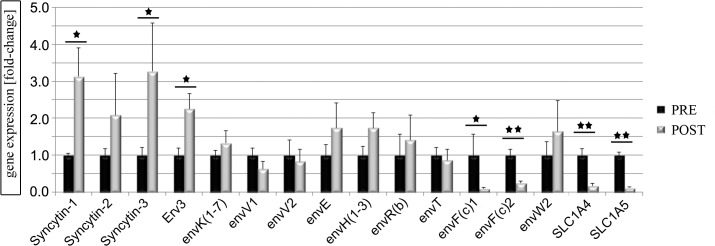
Gene expression profiles of ERV env genes and two receptors SLC1A4 and SLC1A5 after qPCR of cyclists after pre- (PRE) and post-competitive season (POST). Statistical significant genes are in red (PRE = 1-fold) and green (POST = fold). * = P<0.05 and ** = P<0.005.

**Fig 4:**
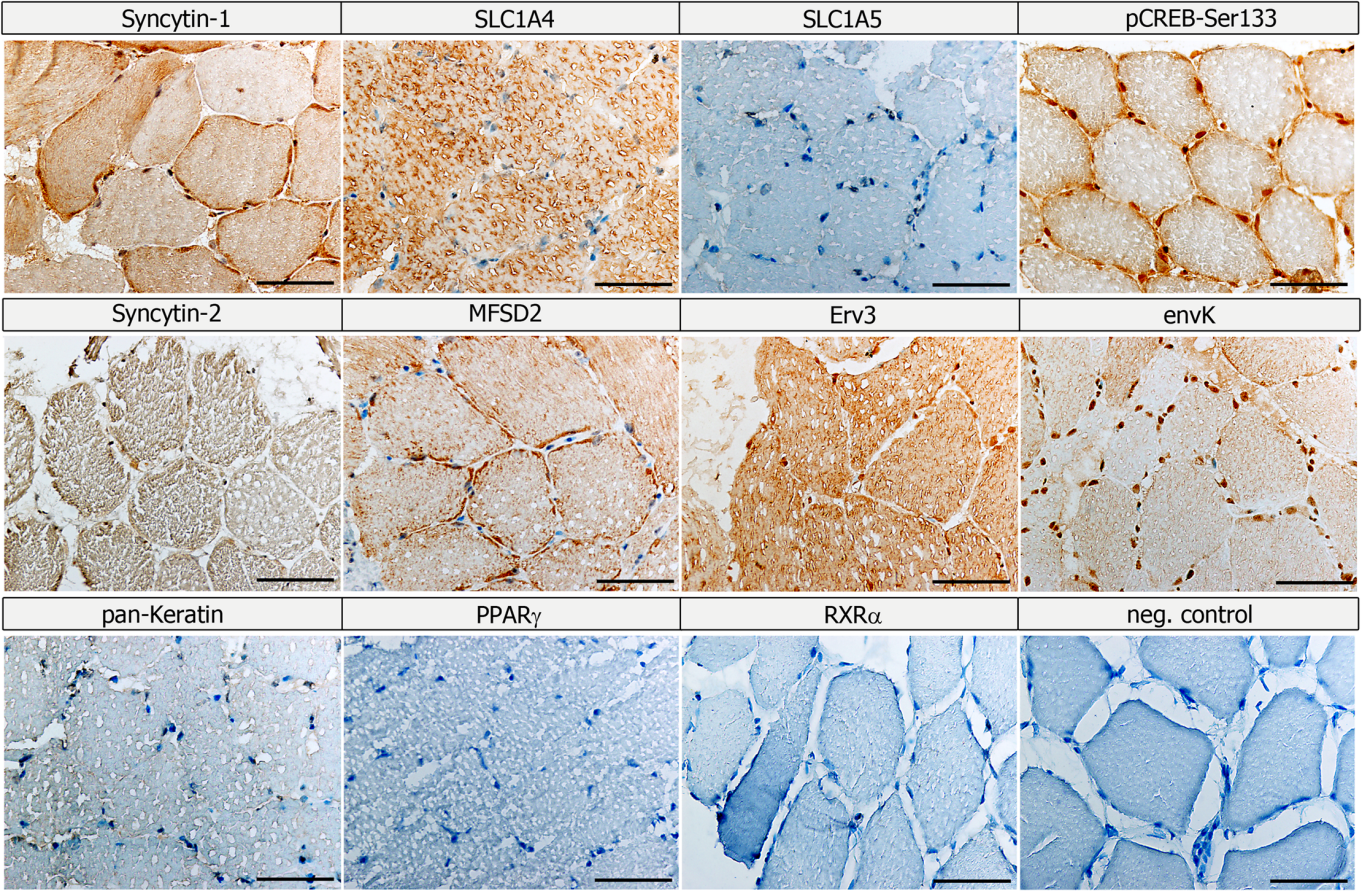
Serial cryocut muscle cross-sections from cyclists after the pre-competitive season demonstrate immuno-localization of different *ERV* env genes and their receptors, transcription factors (pCREB-Ser133, PPARγ and RXRα). An antibody recognizing keratins from skin was used as a negative control for skeletal muscle, as well as a negative (neg.) control without primary antibodies. Bars = 25μm.

**Fig 5:**
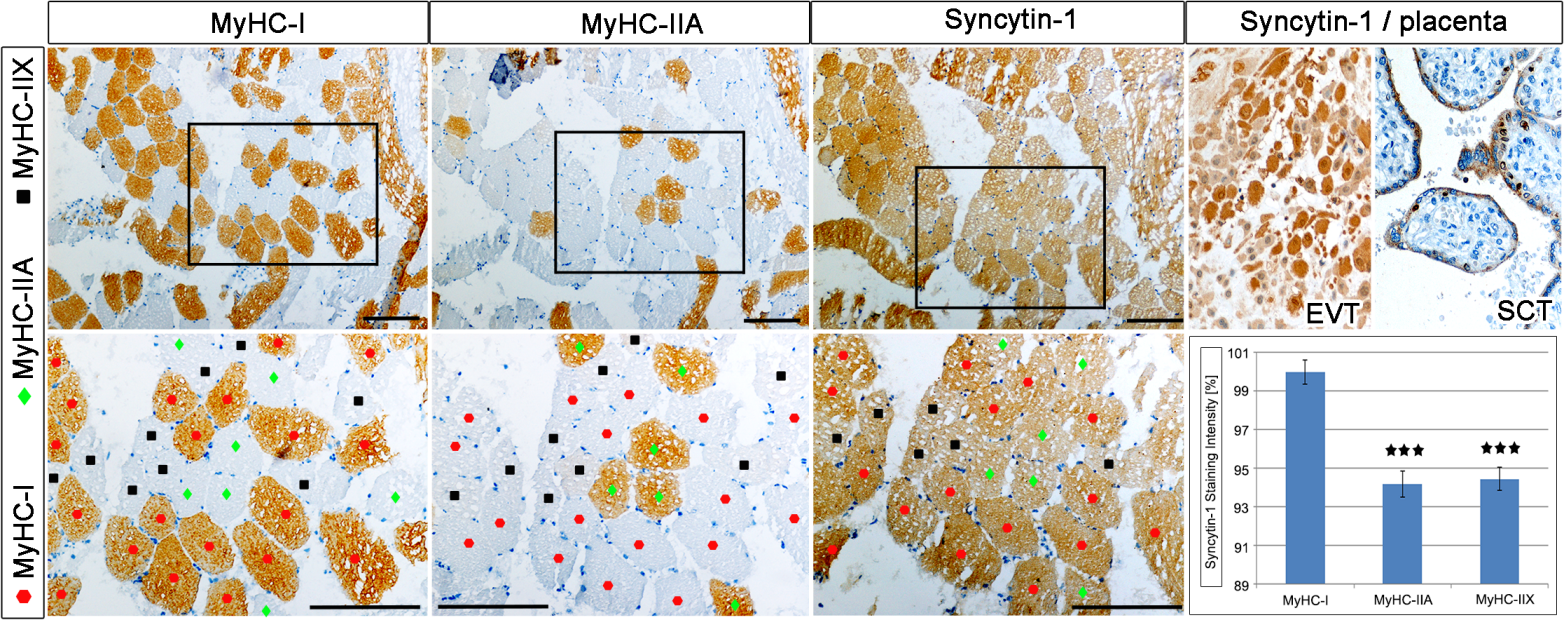
Muscle cross-sections of cyclists after pre-competitive season show consecutive tissue sections with immuno-localization of MyHC-I and MyHC-IIA as well as the fusogenic ERVW-1 env protein Syncytin-1. For comparison of Syncytin-1 protein expression with muscle cells the far right picture shows a positive control of Syncytin-1 immunolocalization on normal third trimester placental tissues [left = extra villous trophoblasts (EVT); right = syncytiotrophoblast (SCT)]. The graph represents a semi-quantitative analysis of Syncytin-1 protein signal intensity measured using ImageJ. The Syncytin-1 expression was then correlated with the fiber types, including MyHC-I (set to 100%), MyHC-IIA and MyHC-IIX. Note that the upper panels show IHC and the lower panels show magnifications of the squares. Color code represents fiber type in the lower panels: red = MyHC-I, green = MyHC-IIA and difference of both is marked black = MyHC-IIX. Bars = 100μm. *** = statistically significant (p< 0.005).

In order to investigate, if human muscle demonstrated significant expression of *ERV env* genes and their receptors, RNA of muscle biopsies from the cyclists was analysed for 22 *ERV env* genes by qPCR and for the Syncytin-1 receptors *SLC1A4* and *SLC1A5* by semi-quantitative PCR (Fig 3 and S1 Table). Only *Syncytin-1*, *Syncytin-3*, and *erv3* showed a significant increase of expression in post-competitive when compared to the pre-competitive season, whereas *envFc1* and *envFc2* showed a significant decrease (Fig 3 and S1 Table). All other *ERV env* genes were not significantly changed in expression. Importantly, *Syncytin-1* and *Syncytin-3* were previously shown as fusogenic proteins participating in cell-cell fusions [5] [54]. The Syncytin-1 cellular receptors *SLC1A4* and *SLC1A5* demonstrated a significant decrease of expression in the post-competitive compared to the pre-competitive season (Fig 3). Further analysis using IHC of ERV env genes, their receptors, as well as Syncytin-1 transcription factors was performed using biopsies from cyclists in the pre-competitive season and demonstrated different cellular locations (Fig 4). Results showed a homogenous Syncytin-1 protein expression throughout myofibers with enrichment at the membrane or sarcolemma. Interestingly, Syncytin-1 myofiber expression was similar in intensity to term placental control tissue, where Syncytin-1 is considered strongly expressed [55] (Fig 5). The Syncytin-1 receptor SLC1A4 only demonstrated positive expression throughout myofibers, whereas the second receptor SLC1A5 was negative. The other fusogenic env gene Syncytin-2, which was not significantly differentially expressed using qPCR, demonstrated positive expression throughout myofibers, whereas its receptor MFSD2 was mainly enriched at the sarcolemma (Fig 4). We were not able to analyze Syncytin-3 expression due to no currently available antibody. The erv3 env protein showed strong positive protein expression throughout the myofiber, whereas, interestingly envK expression localized at the myonuclei and nuclei of SCs. Examining protein expression of Syncytin-1 transcription factors in myofibers demonstrated a unique pCREB-Ser133 protein expression at the basal lamina of the SCs as well as nuclear SCs and myonuclear expression (Fig 4). In contrast the Syncytin-1 transcription factors PPARγ and RXRα, showed no expression in myofibers supporting no regulatory role. According to the literature, PPARγ was in contrast to PPARδ very lowly expressed in healthy skeletal muscle [56].

Due to strong protein expression of Syncytin-1 in myofibers, we asked the question which kind of myofibers were Syncytin-1 positive. Muscle biopsies of the cyclists from the pre-competitive season were immunolocalized for MyHC-I and MyHC-IIA as well as for Syncytin-1 on serial, consecutive sections. Considering the contractile speed of muscle fibers, MyHC-I represent slow or type I fibers whereas MyHC-IIA and IIX represent fast or type II fibers (human skeletal muscle does not express MyHC-IIB). Results showed that the majority of myofiber types were MyHC-I and less were the MyHC-IIA class. The remaining non-MyHC-I and non-MyHC-IIA represented MyHC-IIX positive myofibers (Fig 5). A further comparison of Syncytin-1 protein expression between the different types of myofibers was performed semi-quantitatively. Results demonstrated that Syncytin-1 expression of type I MyHC-I myofibers was significantly stronger in intensity when compared with type II MyHC-IIA and IIX myofibers (Fig 5).

**Fig 6:**
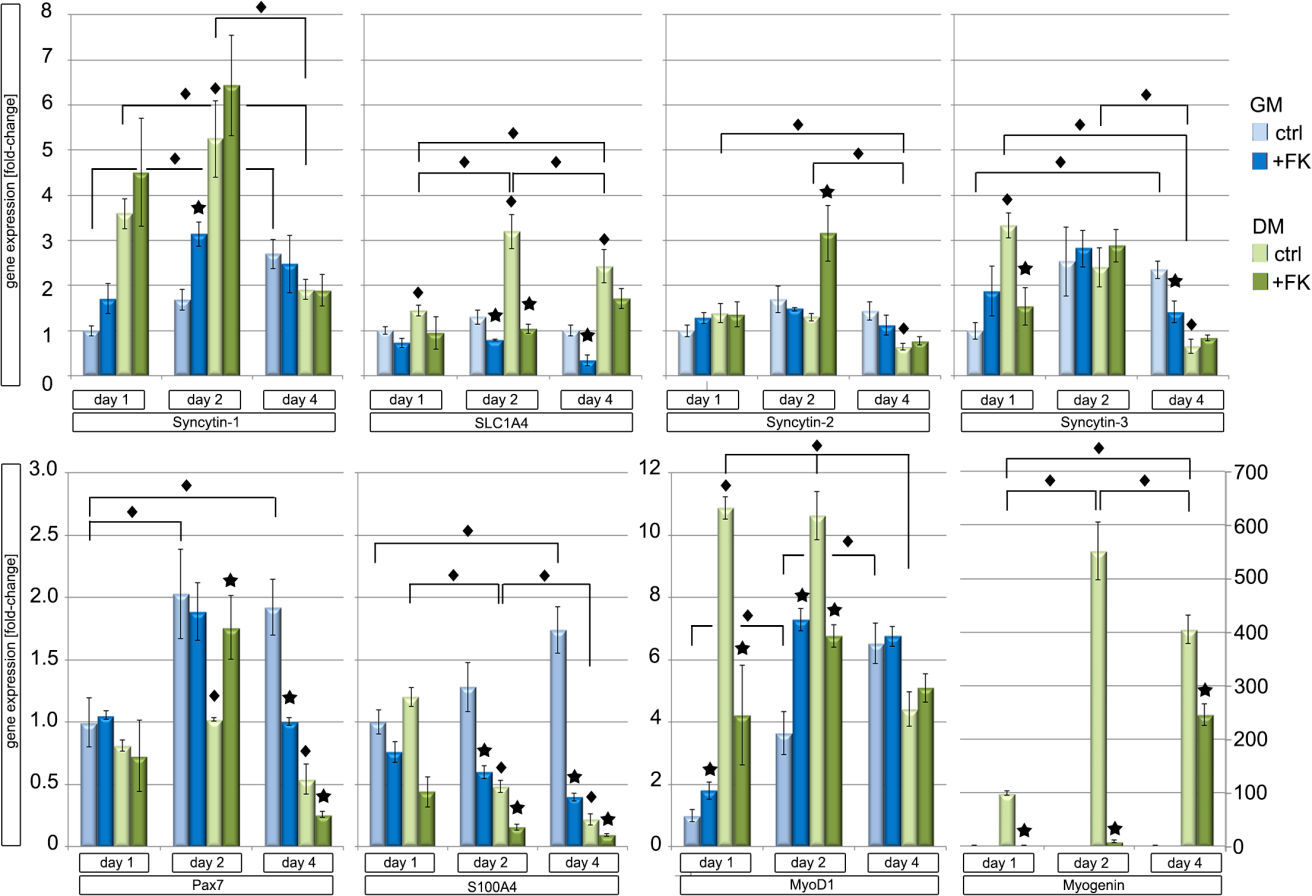
Gene expression profiles of Syncytin-1, -2, -3, and receptor SLC1A4 as well as different muscle specific genes by qPCR and PCR of human primary myoblasts grown for 1, 2 and 4 days in growth medium (GM) and differentiation medium (DM). The value of each gene for day 1 in GM was set as 1. Diamond: Significances (P<0.05) comparing GM and DM at different days; *: significant differences (P<0.05) between Forskolin and no Forskolin addition.

**Fig 7:**
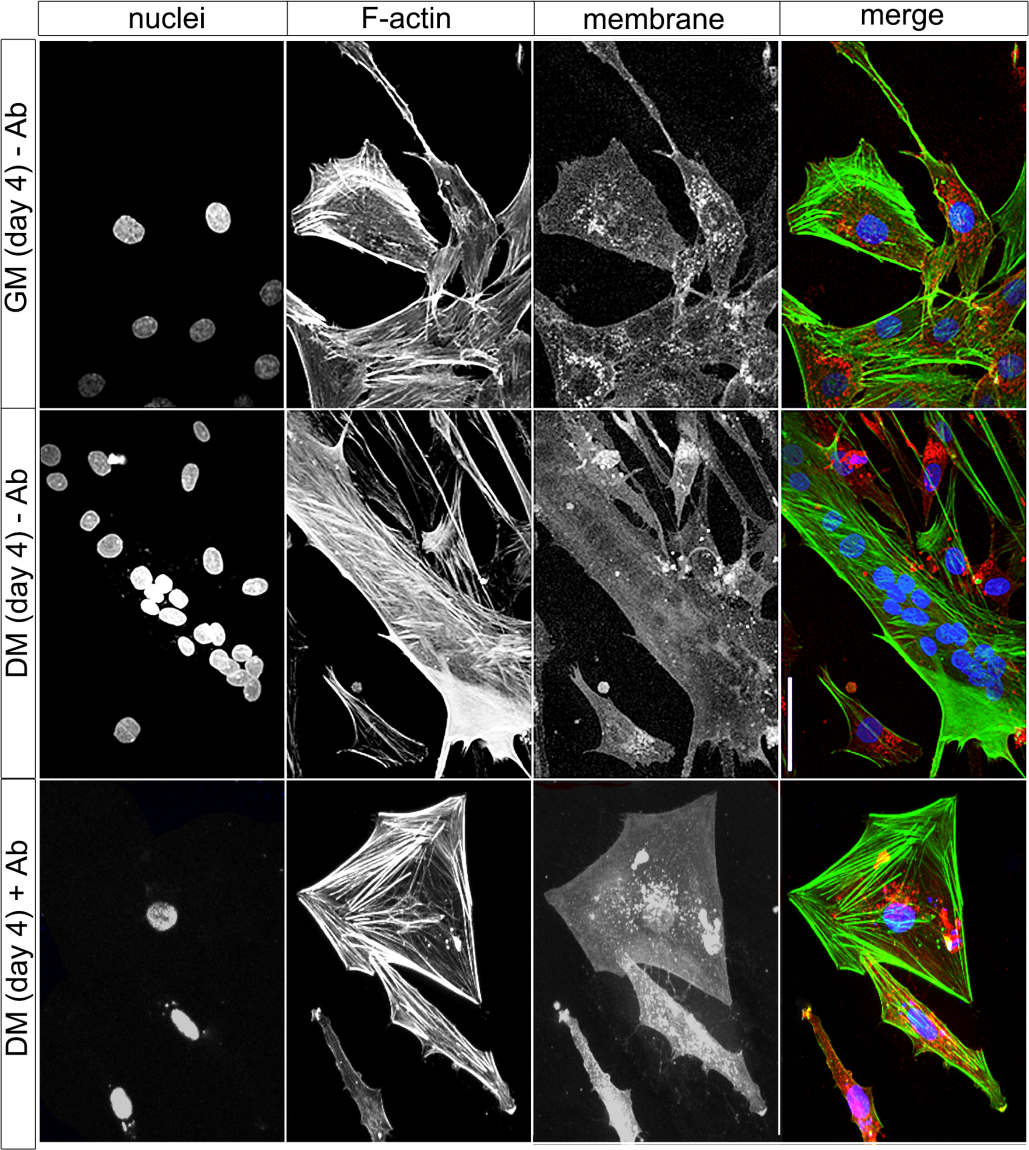
Immunofluorescence of human primary myoblasts in culture with GM and DM with or without anti-Syncytin-1 (-/+ Ab) for 4 days and analysis by confocal microscopy for nuclei (Hoechst 33342), F-actin (Phalloidin Alexa488), membrane (wheat germ agglutinin Alexa594) and the merge with all. Note the multinucleated myofibre in DM. DM composite of 96 z-stacks and GM of 17 z-stacks. Bars = 50μm.

Gene expression of *ERV env* and their receptors were verified using cultured human primary myoblasts, fractionated from muscle biopsies of non-cyclists (Fig 6 and S2 Table). Primary myoblasts were grown in growth media (GM) or differentiation media (DM) for 1–4 days. Importantly, myoblast cells grown in GM demonstrated a 13.4-fold increase of growth at day 4 compared to day 1; on the other hand no significant growth occurred in DM supporting differentiation (data not shown). Regarding gene expression, significant differences of *ERV env* genes and their receptors were observed between GM and DM and showed some similarities to the gene expression of muscle biopsies of cyclists from the pre- and post-competitive season. Although, *Syncytin-1* expression increased in GM from day 1 to day 4 (2.8-fold), a further increase of induction was observed when the cells were switched to DM from day 1 (3.5-fold) till day 2 (5.1-fold) but then decreased until day 4 (2.8-fold). In line with *Syncytin-1* its receptor *SLC1A4* also showed a significant induction of expression from day 1 to day 2 (3.1-fold) in DM (Fig 6). The other putative fusogenic gene *Syncytin-3* showed an early strong induction at day 1 (3.2-fold) in DM, and then decreased in a stepwise manner till day 4 (0.7-fold) (Fig 6). Also examined were several muscle specific genes using cultured human primary myoblasts. For example, *PAX7* showed a significant 2.0-fold induction in GM after day 2 and day 4 but a repression to 0.5-fold on day 4 in DM, supporting the presence of SC growth. As expected expression of *MyoD1* increased using GM but even further increased in DM from day 1 (11-fold) till day 2 (10.2-fold), supporting a role in myoblast growth and differentiation. Importantly, *myogenin* showed the highest induction in DM from day 1 (100-fold) till day 2 (550-fold) and then leveled in expression at day 4 (400-fold), further supporting myoblast differentiation into myotubes. We also checked for the presence of the fibroblast specific marker gene *S100A4*, which showed an induction of expression in GM but a profound reduction by day 2 and 4 in DM (Fig 6). Finally, a microscopic analysis was performed using immunofluorescence of primary human myoblasts cultured in GM and then DM, which clearly demonstrated differentiation of myoblasts into myotubes solely occurring in DM (Fig 7). Specifically differentiated myotubes demonstrated multiple nuclei at day 4 in culture. Co-staining for F-actin also showed an induction of expression in multinucleated myotubes in DM (Fig 7). Furthermore, when a polyclonal antibody specific for Syncytin-1 was incubated directly with myoblasts in DM media for 4 days, myoblast fusion was entirely abrogated (Fig 7, S1 Fig). This result supports Syncytin-1 as an essential protein mediating myoblast cell fusion. Taken together, all of the above findings demonstrate that upon incubation of primary myoblasts in DM, a differentiation and fusion into myotubes occurred, but which could be halted when Syncytin-1 was functionally blocked.

We further tested the influence of Forskolin on myoblast growth and differentiation of primary myoblasts in culture (Fig 6 and S2 Table). Forskolin is a known adenylate cyclase/cAMP and Syncytin-1/cell fusion activator [57]. However, we observed that Forskolin inhibited differentiation of primary myoblasts into myotubes. Thus, no detection of myotubes was seen micoscopically with Forskolin treatment of myotubes (data not shown). Results showed that Forskolin treated myoblasts in DM compared to the control significantly down regulated muscle specific genes (p = 0.05) (Fig 6 and S2 Table). For example, in comparison to control *myogenin* expression decreased throughout day 1 and 2 in DM plus Forskolin and only induced at day 4. *MyoD1* expression levels also decreased on day 1 and 2 (1.5–2.5-fold). In contrast, in GM plus Forskolin *MyoD1* was up-regulated in myoblasts at day 1 and 2, suggesting different roles in cAMP signaling during muscle growth and differentiation. On day 2 *PAX7* showed a significant 1.7-fold increase of gene expression in DM plus Forskolin, suggesting a role of cAMP signaling in SCs. We also investigated the expression of the fusogenic *ERV env* genes *Syncytin-1*, *-2* and *-3* along with *SLC1A4* of primary myoblasts treated with Forskolin (Fig 6). *Syncytin-1* expression was induced on day 1 and 2 in GM and DM, *Syncytin-2* was activated 3.1-fold on day 2 in DM. In contrast to Syncytin-1, its receptor *SLC1A4* was inhibited in the presence of Forskolin in GM and DM, suggesting that the lack of myoblast cell fusion could be linked with the level of receptors. Additionally, Forskolin inhibited expression of *Syncytin-3* on day 1 in DM. Taken together the above results suggest various regulations of ERV *env* genes following cAMP signalling.

## Discussion

Early studies from extensor *digitorum longus* muscles of swimming rats showed that the mean cross-sectional area of muscle fibers was unaltered, but the mean length of capillary per unit volume of muscle and number of nuclei per unit volume of muscle was increased [58]. Similarly in our study with muscles from cyclists comparing pre- and post-competitive seasons the number of myonuclei/mm fiber length increased (2.25-fold), however with no change of the muscle fiber cross-sectional area or the myonuclear length. Additionally, the fiber area per nucleus significantly decreased by 2.4-fold in the post-competitive season. Thus, as cell fusion proceeds and the number of nuclei increases within a muscle fiber, the internal fiber area per nucleus must decrease in order to maintain a constant fiber cross-sectional area, which is necessary to maintain proper skeletal muscle homeostasis and tissue integrity. It is interesting to note that other human studies in part demonstrated differences, which most likely could be attributed to the type of sport and biopsy localization. For example, when *vastus lateralis* biopsies were analyzed from fifteen individuals who performed 3-times/week resistance leg training, a significant increase in muscle fiber area occurred after 90 days along with the myonuclear domain and the number of SC [59]. In contrast, the authors found no difference in the myonuclear number. Further comparisons from the literature involving power lifters (n = 10), who trained 4-6-times per week, when compared to controls (n = 6) showed an increased number of myonuclei/fiber from trapezius muscles, further indicating a higher cell fusion rate in muscle sport activities [60]. This study also showed an increased number of SC in muscle fibers compared to controls.

It is known that an increase of SC numbers is essential for the maintenance and repair of muscle function. Using skeletal muscles of rats it was shown that different intensities and durations of training (treadmill) had no influence on the mean fiber area and myonuclei per fiber. However, the SC pool increased in rats who trained with a higher intensity rather than an increased duration [61]. Another study involving mice exercising with moderate intensity for 8 weeks on a treadmill also showed an increase of SC [62]. Increases of the SC amount in the skeletal muscles have also been reported with over 14 weeks of endurance training in healthy older men [63, 64]. Considering the SC amount, our study showed that a 3.1-fold decrease of SC occurred in muscles of cyclists comparing the post- with the pre-competitive season. This result speaks for proliferation of SCs during moderate training in the pre-competitive season, in contrast to a possible SC exhaustion due to increased cell fusion during the competitive season.

Many proteins have been found to be essential in myoblast fusion, like myogenin [65], DOCK1 [66], Rac1, Cdc42 [67] and N-WASP [68] [69]. Especially the ELMO-DOCK1-Rac1-pathway, which regulates the actin cytoskeleton, has an essential role in myoblast fusion. New studies linked transmembrane proteins like brain-specific angiogenesis inhibitors (BAI) and Myomaker to murine myoblast fusion. For example, one study identified BAI1 as a receptor for phosphatidylserine presented by apoptotic cells as crucial for myoblast fusion during muscle repair [70]. An additional family member, BAI3 was also found necessary for myoblast fusion by its interaction with ELMO [71]. Another driver for myoblast fusions was the murine transmembrane protein Myomaker, which was found to induce cell fusions, when only expressed in one partner cell [72]. Subsequently, a yet unknown receptor on the recipient cell must be necessary for myoblast fusion events. The actin-cytoskeleton is also essential for cell fusions. In *Drosophila*, the protein complex FuRMAS is a signaling center at cell-cell contact sites, including F-actin, cell adhesion and signaling proteins. Especially, F-actin accumulation and branching is one prerequisite for myoblast fusion in *Drosophila* [69]. In another *Drosophila* study, two fusing muscle cells showed F-actin foci along the membrane at the site of fusion, with invading finger-like cell protrusions occurring from one cell to another [73]. Although we did not detect myoblast fusions “*in statu nascendi”*; differences were observed with F-actin polymerization of primary myoblast cells grown in GM and DM after 4 days in culture (Fig 7). For example, the mononuclear myoblasts in GM showed high F-actin polymerization at parts of the cell membrane, whereas multinuclear myotubes showed a more uniform F-actin polymerization.

Our present research findings implicate that the ERV *env* genes, *Syncytin-1* and *Syncytin-3* are involved in human myoblast fusion, for example the over 3-fold higher level of expression observed for both genes in fused muscle fibers of cyclists at the post-competitive season and the detection of Syncytin-1 at the sarcolemma using IHC (Fig 4). As noted earlier in the literature, *Syncytin-1* was found significantly elevated in muscle biopsies of patients with motor neuron disease compared to *Syncytin-1* expression in muscles of control individuals [74]. The latter study implicated that increased levels of *Syncytin-1* in muscles from diseased patients was linked with oxidative stress and cytotoxicity. A recent study also demonstrated that the Syncytin-1 protein localized to membranes of connecting human primary cultured myoblasts and partially co-localized with caveolin-3 in myogenin-positive and negative cells [75]. In the same study antisense primers against Syncytin-1 inhibited cell fusion of cultured myoblasts. Further confirmation for a role of Syncytins in myoblast cell fusion stems from our *in vitro* primary myoblast cultures, where myoblasts in DM showed a significant induction of *Syncytin-1* after day 1 and 2. Importantly, our results that cell fusion of myoblasts were blocked using an antibody targeting Syncytin-1 protein points to an essential role of this membrane protein in myoblast differentiation. Its receptor *SLC1A4* was also induced on day 2 and 4 in DM. Additionally, *Syncytin-3* expression was induced earlier on day 1 in DM. Overall we predict that myoblast differentiation was maximal after day 1 and day 2 and with a reduction of gene expression by day 4 along with the presence of multinucleated myotubes supports differentiation was complete. It is known that Syncytin proteins require cellular receptors to mediate cell fusions, like SLC1A4 and SLC1A5 (for Syncytin-1) and MFSD2a (for Syncytin-2), however a cellular receptor for Syncytin-3 is not known so far. Northern blots previously demonstrated that *SLC1A5* (also called receptor for RD114/type D retrovirus) was mainly expressed in a variety of tissues containing skeletal muscles [76]. Since we only detected the SLC1A4 and not SLC1A5 protein in muscle from pre-competitive season, supports translational regulations for SLC1A5. Therefore in skeletal muscles of cyclists, SLC1A4 appears to be the primary transporter for both glutamate and neutral amino acids and the main receptor of Syncytin-1 for mediating cell fusions. Additionally, a lower requirement for amino acids after the 8 month long competitive season could contribute to the abrupt decrease of *SLC1A4* gene expression. Taken together, our results implicate that both ERV env genes along with their receptors may be responsible for active myoblast fusion *in vivo* and *in vitro*.

An induced protein kinase A pathway characterized through elevated cAMP and pCREB-Ser133 has been shown to be responsible for the activation of Syncytin-1 in placental trophoblasts [77], choriocarcinoma cells [78], endometrial carcinoma [79] and pituitary adenomas [80] with different cellular outcomes. Strong pCREB-Ser133 signals were found at muscle fibers of the cyclists after the pre-competitive season using IHC (Fig 4). Interestingly, like envK, pCREB-Ser133 postively localized to the myonuclei and SC nuclei of myofibers of cyclists in the pre-competitive season (Fig 4), pointing to a role in the SC regulation. Using our primary myoblast cell cultures *PAX7* gene expression was significantly induced in GM at day 2 and also following Forskolin treatment in DM, further implicating cAMP regulation of SCs. A study of rat SC showed that an inhibition of protein kinase A (and protein kinase C) induced differentiation (cell fusion) without affecting proliferation [81]. This suggests protein kinase A negatively regulates muscle fusion. Therefore, our study points to a unique role of the protein kinase A-pathway (pCREB-Ser133) regulation of SCs possibly inducing a shift towards a more undifferentiated state. This could help to explain our results of a higher SC content and reduced cell fusion during the pre- compared to the post-competitive season.

In search of pro-myogenic compounds using Zebrafish embryo cultures, Forskolin at a 50μM concentration enhanced mouse SC proliferation and together with bFGF and a GSK3ß inhibitor, induced skeletal differentiation of human induced pluripotent stem cells [82]. Interestingly, the latter study found no induction of cell fusion in cell culture with Forskolin. In contrast to Xu et al. [82] who showed no change of cell fusion with Forskolin, our results demonstrate that Forskolin inhibited cell fusion of primary myoblasts measured over 4 days. Similar to other cell culture studies [77–80], we also found that *Syncytin-1* gene expression was significantly induced in primary myoblasts with Forskolin on day 1 and 2 in DM and GM. However, the inhibition of *SLC1A4*, *MyoD1* and *myogenin* with Forskolin along with our microscopic findings supports no induction of cell fusion. Comparable to a study with Forskolin and human pituitary adenoma cells, Forskolin induced *Syncytin-1* and also did not result in cell fusions [80]. This is in contrast to human primary placental trophoblasts (not choriocarcinoma cells), which showed high cell fusions and Syncytin-1 induction after Forskolin treatment [11, 83]. In agreement with our findings showing inhibition of primary myoblast fusion with Forskolin, another study showed that protein kinase A activation through elevated cAMP levels inhibited skeletal myogenesis by phosphorylating and inactivating myocyte enhancer factor 2D [84].

**Fig 8:**
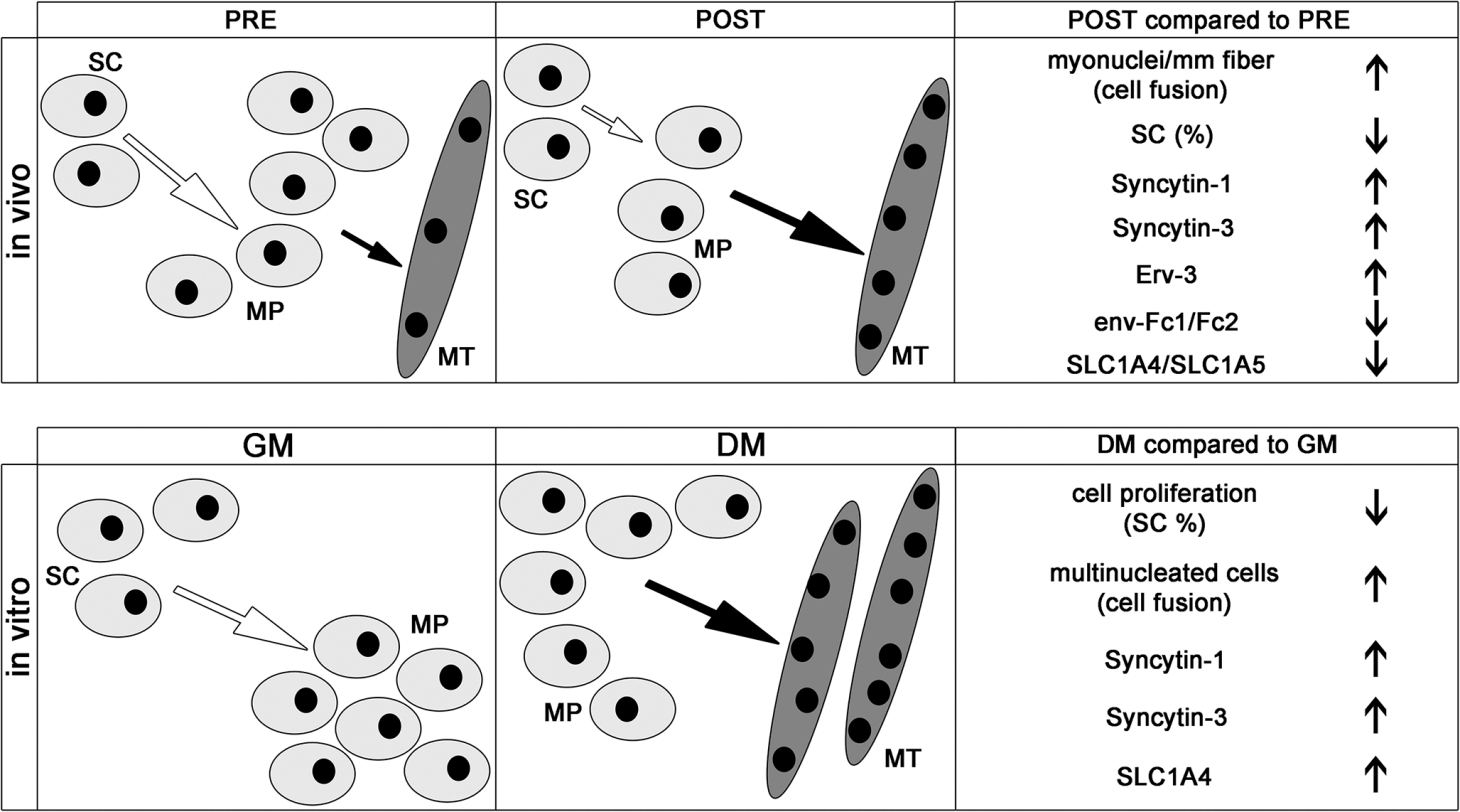
Schematic model showing the links between the significant changes of muscle-specific attributes with the expression of ERV env genes, their receptors and muscle specific genes relating to cell fusion occurring *in vivo* (biopsies from cyclists at the pre- and post- competitive seasons) and *in vitro*. The top represents the muscle differentiation in cyclists from pre- (PRE) to post-competitive season (POST), whereas the bottom symbolizes the myoblast cultures proliferating in growth media (GM) or differentiating to myotubes in differentiation media (DM). Additionally, since SCs and myonuclei showed positive expression for protein kinase A activated pCREB-Ser133 (Fig 4) and treatment of primary myoblast cultures with the cAMP stimulator Forskolin did not promote myoblast cell fusion (Fig 6), we predict that cAMP may be important for regulating SCs. SC = satellite cells; MP = muscle progenitors; MT = myotubes; PRE = pre-competition; POST = post-competition; GM = growth media; DM = differentiation media; arrow up = significantly up-regulated and arrow down = significantly down-regulated.

In summary, based on our results, we present a model demonstrating the link between the process of muscle differentiation in human cyclists *in vivo* and human myoblasts *in vitro* (Fig 8). In the future it will also be important to unravel the diverse functional roles of cAMP signaling and ERV gene regulations during cell fusion in different cellular types including muscle cell progenitors.

## Supporting Information

S1 Fig Inhibition of primary myoblasts fusion after treatment with anti-Syncytin-1. Panel shows fluorescence imaging of human primary myoblasts cultured in DM, without or treated with anti-Syncytin-1 (Ab) for 4 days and then analysed using a fluorescent microscope and computer software. Merged images show nuclei (Hoechst 33342, blue) and cell membrane (wheat germ agglutinin Alexa594, red). White arrows represent multinucleated myofibres in DM with no antibody.
(TIF)

S1 Table ERV env gene expression in muscle biopsies from cyclists. (DOCX)

S2 Table Muscle specific gene expression of primary muscle cell cultures with and without Forskolin. (DOCX)
