# Supplementary figures and images for: Long-Term Endurance Exercise in Humans Stimulates Cell Fusion of Myoblasts along with Fusogenic Endogenous Retroviral Genes In Vivo
Source: PLoS One. 2015 Jul 8;10(7):e0132099. doi: 10.1371/journal.pone.0132099 (PMC4495930; doi:10.1371/journal.pone.0132099)

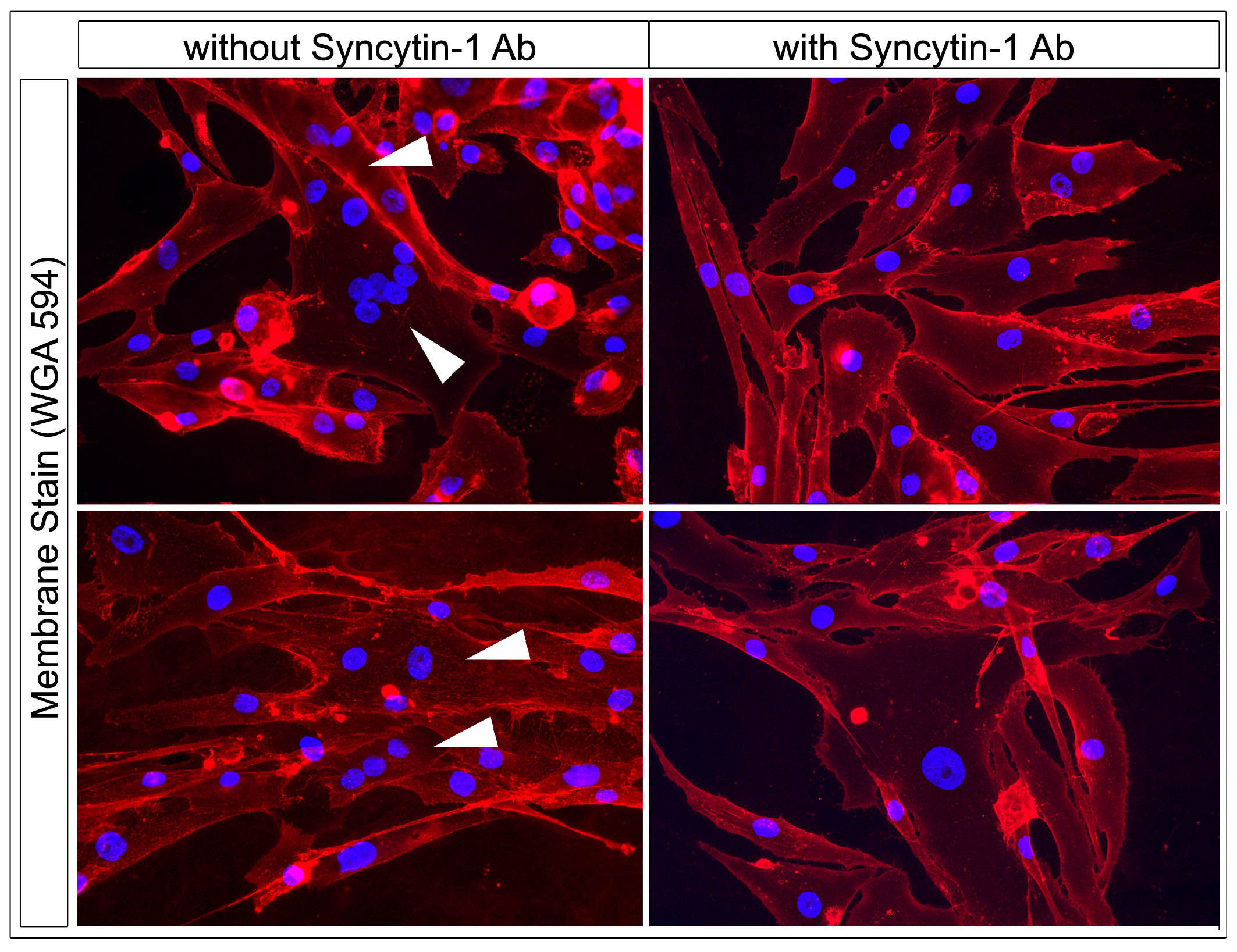

Supplement: S1 Fig — Panel shows fluorescence imaging of human primary myoblasts cultured in DM, without or treated with anti-Syncytin-1 (Ab) for 4 days and then analysed using a fluorescent microscope and computer software. Merged images show nuclei (Hoechst 33342, blue) and cell membrane (wheat germ agglutinin Alexa594, red). White arrows represent multinucleated myofibres in DM with no antibody. (TIF) [file pone.0132099.s001.tif]
