# Supplementary material for: Long-Term Endurance Exercise in Humans Stimulates Cell Fusion of Myoblasts along with Fusogenic Endogenous Retroviral Genes In Vivo
Source: PLoS One. 2015 Jul 8;10(7):e0132099. doi: 10.1371/journal.pone.0132099 (PMC4495930; doi:10.1371/journal.pone.0132099)
Supplement: S1 Table — (DOCX) [file pone.0132099.s002.docx]

Supplemental Table 1: ERV env gene expression in muscle biopsies from cyclists.

|  | ***Syncytin-1*** | ***Syncytin-2*** | ***Syncytin-3*** | ***Erv3*** | ***envK(1-7)*** | ***envV1*** | ***envV2*** | ***envE*** | ***envH(1-3)*** | ***envR(b)*** | ***envT*** | ***envF(c)1*** | ***envF(c)2*** | ***envW2*** |
| --- | --- | --- | --- | --- | --- | --- | --- | --- | --- | --- | --- | --- | --- | --- |
| **PRE mean** | **64.53** | **2.00** | **32.37** | **44.30** | **108.81** | **1.19** | **0.60** | **13.36** | **72.30** | **0.05** | **25.18** | **1.93** | **21.63** | **1.77** |
| sem | ±3.24 | ±0.37 | ±6.79 | ±8.38 | ±14.82 | ±0.23 | ±0.25 | ±3.86 | ±17.03 | ±0.03 | ±5.17 | ±1.10 | ±3.53 | ±0.66 |
| **POST mean** | **201.68** | **4.17** | **105.90** | **100.17** | **144.47** | **0.75** | **0.51** | **23.35** | **125.86** | **0.08** | **21.98** | **0.20** | **5.71** | **2.93** |
| sem | ±49.53 | ±2.25 | ±42.33 | ±17.74 | ±37.28 | ±0.25 | ±0.20 | ±8.90 | ±29.90 | ±0.04 | ±7.48 | ±0.05 | ±1.00 | ±1.44 |
| **p-value** | **0.041** | 0.912 | **0.026** | **0.033** | 0.971 | 0.105 | 0.739 | 1.000 | 0.340 | 0.165 | 0.315 | **0.020** | **0.001** | 0.912 |

Values for Pre and Post mean +/- sem are molecules/ ng cDNA. P-values in bold were significant (<0.05).
