# Supplementary material for: Long-Term Endurance Exercise in Humans Stimulates Cell Fusion of Myoblasts along with Fusogenic Endogenous Retroviral Genes In Vivo
Source: PLoS One. 2015 Jul 8;10(7):e0132099. doi: 10.1371/journal.pone.0132099 (PMC4495930; doi:10.1371/journal.pone.0132099)
Supplement: S2 Table — (DOCX) [file pone.0132099.s003.docx]

|  |  | **Syn1** | **SLC1A4** | **Syn2** | **Syn3** | **PAX7** | **MyoD1** | **S100A4** | **myogenin** |
| --- | --- | --- | --- | --- | --- | --- | --- | --- | --- |
| **control** | **day1 GM** | **1.00** | **1.00** | **1.00** | **1.00** | **1.00** | **1.00** | **1.00** | **1.00** |
|  | sem | 0.11 | 0.08 | 0.13 | 0.19 | 0.20 | 0.18 | 0.09 | 0.14 |
|  | **day2 GM** | **1.69** | **1.30** | **1.69** | **2.53** | **2.03** | **3.63** | **1.28** | **0.76** |
|  | sem | 0.23 | 0.16 | 0.29 | 0.76 | 0.36 | 0.69 | 0.19 | 0.11 |
|  | p-value (day1 GM) | 0.05 | 0.13 | 0.27 | 0.13 | 0.13 | 0.05 | 0.28 | 0.28 |
|  | **day4 GM** | **2.70** | **1.01** | **1.44** | **2.35** | **1.92** | **6.52** | **1.74** | **1.49** |
|  | sem | 0.33 | 0.11 | 0.21 | 0.20 | 0.22 | 0.65 | 0.18 | 0.27 |
|  | p-value (day1 GM) | 0.05 | 0.83 | 0.28 | 0.05 | 0.05 | 0.05 | 0.05 | 0.13 |
|  | **day1 DM** | **3.60** | **1.44** | **1.38** | **3.33** | **0.81** | **10.87** | **1.20** | **99.32** |
|  | sem | 0.33 | 0.13 | 0.21 | 0.28 | 0.04 | 0.37 | 0.08 | 3.90 |
|  | p-value (day1 GM) | 0.05 | 0.05 | 0.28 | 0.05 | 0.51 | 0.05 | 0.13 | 0.05 |
|  | **day2 DM** | **5.25** | **3.19** | **1.29** | **2.40** | **1.02** | **10.63** | **0.48** | **552.30** |
|  | sem | 0.85 | 0.38 | 0.09 | 0.44 | 0.01 | 0.77 | 0.05 | 53.92 |
|  | p-value (day1 GM) | 0.05 | 0.05 | 0.05 | 0.05 | 0.51 | 0.05 | 0.05 | 0.05 |
|  | p-value (day1 DM) | 0.13 | 0.05 | 0.83 | 0.13 | 0.05 | 0.51 | 0.05 | 0.05 |
|  | **day4 DM** | **1.91** | **2.43** | **0.65** | **0.65** | **0.54** | **4.42** | **0.22** | **404.96** |
|  | sem | 0.23 | 0.37 | 0.08 | 0.15 | 0.12 | 0.54 | 0.04 | 26.80 |
|  | p-value (day1 GM) | 0.05 | 0.05 | 0.13 | 0.27 | 0.13 | 0.05 | 0.05 | 0.05 |
|  | p-value (day1 DM) | 0.05 | 0.05 | 0.05 | 0.05 | 0.13 | 0.05 | 0.05 | 0.05 |
| **with Forskolin** | **day1 GM** | **1.71** | **0.73** | **1.28** | **1.88** | **1.06** | **1.81** | **0.76** | **0.13** |
|  | sem | 0.33 | 0.09 | 0.12 | 0.55 | 0.04 | 0.28 | 0.08 | 0.02 |
|  | p-value (day1 GM) | 0.13 | 0.13 | 0.28 | 0.13 | 0.51 | 0.05 | 0.13 | 0.05 |
|  | **day2 GM** | **3.15** | **0.79** | **1.48** | **2.83** | **1.89** | **7.29** | **0.60** | **0.29** |
|  | sem | 0.26 | 0.02 | 0.04 | 0.41 | 0.23 | 0.36 | 0.05 | 0.04 |
|  | p-value (day1 GM) | 0.05 | 0.51 | 0.28 | 0.28 | 0.05 | 0.05 | 0.05 | 0.05 |
|  | p-value (day2 GM) | 0.05 | 0.05 | 0.51 | 0.83 | 0.51 | 0.05 | 0.05 | 0.05 |
|  | **day4 GM** | **2.48** | **0.35** | **1.12** | **1.41** | **1.00** | **6.76** | **0.40** | **0.15** |
|  | sem | 0.64 | 0.12 | 0.23 | 0.24 | 0.03 | 0.31 | 0.03 | 0.05 |
|  | p-value (day1 GM) | 0.28 | 0.05 | 0.83 | 0.51 | 0.38 | 0.05 | 0.05 | 0.51 |
|  | p-value (day4 GM) | 0.83 | 0.05 | 0.28 | 0.05 | 0.05 | 0.51 | 0.05 | 0.05 |
|  | **day1 DM** | **4.51** | **0.95** | **1.37** | **1.53** | **0.73** | **4.23** | **0.44** | **1.91** |
|  | sem | 1.20 | 0.35 | 0.28 | 0.41 | 0.29 | 1.61 | 0.12 | 0.50 |
|  | p-value (day1 GM) | 0.05 | 0.83 | 0.83 | 0.51 | 0.51 | 0.05 | 0.18 | 0.05 |
|  | p-value (day1 DM) | 0.83 | 0.28 | 0.83 | 0.05 | 0.51 | 0.05 | 0.05 | 0.05 |
|  | **day2 DM** | **6.43** | **1.05** | **3.16** | **2.88** | **1.76** | **6.76** | **0.16** | **9.46** |
|  | sem | 1.12 | 0.10 | 0.61 | 0.37 | 0.26 | 0.35 | 0.03 | 2.17 |
|  | p-value (day1 GM) | 0.05 | 0.13 | 0.05 | 0.13 | 0.05 | 0.05 | 0.05 | 0.05 |
|  | p-value (day1 DM) | 0.28 | 0.51 | 0.05 | 0.13 | 0.05 | 0.51 | 0.05 | 0.05 |
|  | p-value (day2 DM) | 0.51 | 0.05 | 0.05 | 0.28 | 0.05 | 0.05 | 0.05 | 0.05 |
|  | **day4 DM** | **1.90** | **1.71** | **0.78** | **0.84** | **0.26** | **5.09** | **0.09** | **245.94** |
|  | sem | 0.35 | 0.22 | 0.09 | 0.06 | 0.03 | 0.46 | 0.01 | 20.19 |
|  | p-value (day1 GM) | 0.51 | 0.05 | 0.05 | 0.05 | 0.05 | 0.05 | 0.05 | 0.05 |
|  | p-value (day1 DM) | 0.05 | 0.13 | 0.13 | 0.05 | 0.05 | 0.51 | 0.05 | 0.05 |
|  | p-value (day4 DM) | 0.83 | 0.13 | 0.28 | 0.27 | 0.05 | 0.28 | 0.05 | 0.05 |

Supplemental Table 2: Muscle specific gene expression of primary muscle cell cultures with and without Forskolin. Syn1 = Syncytin-1; Syn2 = Syncytin-2; Syn3 = Syncytin-3.
